# Return Customers: Foraging Site Fidelity and the Effect of Environmental Variability in Wide-Ranging Antarctic Fur Seals

**DOI:** 10.1371/journal.pone.0120888

**Published:** 2015-03-25

**Authors:** Benjamin Arthur, Mark Hindell, Marthan Bester, Phil Trathan, Ian Jonsen, Iain Staniland, W. Chris Oosthuizen, Mia Wege, Mary-Anne Lea

**Affiliations:** 1 Institute for Marine and Antarctic Studies, University of Tasmania, Hobart, Tasmania, Australia; 2 Mammal Research Institute, Department of Zoology and Entomology, University of Pretoria, Pretoria, South Africa; 3 British Antarctic Survey, Cambridge, United Kingdom; 4 Department of Biological Sciences, Macquarie University, Sydney, New South Wales, Australia; Phillip Island Nature Parks, AUSTRALIA

## Abstract

Strategies employed by wide-ranging foraging animals involve consideration of habitat quality and predictability and should maximise net energy gain. Fidelity to foraging sites is common in areas of high resource availability or where predictable changes in resource availability occur. However, if resource availability is heterogeneous or unpredictable, as it often is in marine environments, then habitat familiarity may also present ecological benefits to individuals. We examined the winter foraging distribution of female Antarctic fur seals, *Arctocephalus gazelle*, over four years to assess the degree of foraging site fidelity at two scales; within and between years. On average, between-year fidelity was strong, with most individuals utilising more than half of their annual foraging home range over multiple years. However, fidelity was a bimodal strategy among individuals, with five out of eight animals recording between-year overlap values of greater than 50%, while three animals recorded values of less than 5%. High long-term variance in sea surface temperature, a potential proxy for elevated long-term productivity and prey availability, typified areas of overlap. Within-year foraging site fidelity was weak, indicating that successive trips over the winter target different geographic areas. We suggest that over a season, changes in prey availability are predictable enough for individuals to shift foraging area in response, with limited associated energetic costs. Conversely, over multiple years, the availability of prey resources is less spatially and temporally predictable, increasing the potential costs of shifting foraging area and favouring long-term site fidelity. In a dynamic and patchy environment, multi-year foraging site fidelity may confer a long-term energetic advantage to the individual. Such behaviours that operate at the individual level have evolutionary and ecological implications and are potential drivers of niche specialization and modifiers of intra-specific competition.

## Introduction

Foraging animals are expected to make prudent choices in order to minimise energy expenditure whilst maximising energy intake. The choice of foraging habitat is an important component of this, and various foraging ecology models have sought to describe how these choices might be made. One of the best established models, the Marginal Value theorem [1], predicts that foragers in patchy environments balance their rate of energy intake with the energy expenditure associated with travel, search and prey handling times, and that as energy intake in a particular area declines, foragers should move to other, more profitable areas. While various studies of foraging ecology yield support for such theories [2–4], other descriptions of foraging behaviours apparently seem contradictory. Site fidelity; the return to and re-use of a previously occupied area [5], where reduced patch switching often results, is one such example. Individuals from a range of taxa including mammals [6, 7], birds [8, 9], fish [10] and insects [11] repeatedly return to foraging sites. We may consider such behaviour a form of optimal foraging [12], where the act of remaining faithful to a site delivers an increase in net energy intake, particularly in environments with high resource availability.

The quality of resources, however, is unlikely to be the only factor influencing an animal’s choice of foraging habitat, with the stability and predictability of the resources also likely to play an important role. When habitats are relatively stable, or have predictable spatial and temporal changes in food availability, site fidelity can occur [13]. This is particularly common in terrestrial environments with highly predictable food resources, such as fruiting or flowering trees [14]. However, foraging site fidelity is also documented in marine species, including seabirds [15], pinnipeds [16, 17], turtles [18] and cetaceans [19], which typically rely on what are regarded as unpredictable and patchily distributed prey [20, 21]. If habitat quality is heterogeneous and unpredictable, either spatially or temporally, site fidelity can also present ecological benefits to individuals, such as familiarity with resources [22] or reduced predation risk [23]. For long-lived animals, such as many vertebrate marine predators, the persistence of long-term fidelity (i.e. over months and years) to foraging sites [24, 25] may serve to maximise net energy intake over the individual’s lifetime [26], even if energy intake is not high in all years [27].

The availability of prey resources to marine predators varies through normal atmospheric and oceanic processes, for example in the Southern Ocean, the Southern Annular Mode (SAM) [28], the El Niño Southern Oscillation (ENSO) and the formation and retreat of sea ice [29]. Despite this, just how higher trophic levels will respond to future change remains poorly understood. This is especially important for animals demonstrating strong site fidelity as it raises questions about behavioural plasticity and their ability to respond to future habitat alterations such as those arising from the effects of climate change and the activities of fisheries. Typically, ecologists have viewed foraging behaviour at the population level, treating individuals as ecologically alike [30]. However, it is at the individual level where natural selection operates and, consequently, individual specializations have potential evolutionary (e.g. niche specialization) and ecological (e.g. intra-specific competition) implications for population structure. To reliably assess the importance of behaviours such as individual site fidelity, longitudinal studies are required. Few such studies exist for marine predators, with only a handful seeking to track the same individuals over multiple seasons within the same area [27, 31–34].

Antarctic fur seals (*Arctocephalus gazella*, AFS) are top marine predators that present an ideal model for investigating site fidelity. During the non-breeding austral winter many female AFS undertake wide-ranging migrations or dispersals [35, 36]. During this time, they are free from the constraints of central place foraging [37] associated with provisioning their offspring. These movements, therefore, afford insights into foraging habitat preferences during an unconstrained period. Furthermore, female AFS become pregnant during the winter season when the blastocyst implants [38] and must make judicious choices in regards to maximising their energy intake in the important pre-breeding period. Studies of the foraging behaviour of AFS during the summer breeding season are frequent in the literature and generally demonstrate that animals target specific foraging areas [39–41]; nevertheless, few data exist concerning the degree to which individuals return to these areas in successive trips [42] and no studies have investigated longer term site fidelity over multiple seasons.

We quantified the winter foraging patterns of female AFS over four years between 2008–11 to identify the degree of site fidelity to Southern Ocean foraging habitats. A coordinated, long-term tracking program allowed us to examine site fidelity at two scales: within a year and between years. We examined site fidelity in relation to several remotely-sensed environmental parameters, using long-term oceanic variability (i.e. predictability) as a proxy for productivity and prey availability [27, 43]. We hypothesise that fidelity to foraging areas will be related to resource availability and that this behaviour will confer energetic benefits to the individual. We discuss the possible mechanisms driving foraging site fidelity and the potential ecological and evolutionary implications of this behaviour.

## Methods

### Ethics Statement

All animal handling and experimentation were undertaken with approval from the University of Tasmania Animal Ethics Committee (permit A001134), the University of Pretoria Animal Use and Care Committee (permit AUCC 040827–024) and the joint British Antarctic Survey-Cambridge University Animal Ethics Review Committee (does not issue permit numbers). Considering the very small size of the tags used in this study (see below) and the relatively high rate of recovery at Marion Island (Table 1), the impact of animals in carrying these tags is minimal.

**Table 1:** Sample sizes (number of individual animals and trips) by site and year used to estimate foraging habitat overlap at two temporal scales, within-year (encompasses multiple foraging trips undertaken by an animal in one season) and between-year (animals tracked over multiple years).

Site	Year	GLS model	GLS deployed	GLS recovered	Animals tracked		Trips available^a^		
					Fidelity level		All	Fidelity level	
					Within-year	Between-year^b^		Within-year	Between-year
Marion Island	2008	Mk7	30	20	9	Yes (7)	42	4	18
	2009	Mk7	31	15	8	Yes (7)	25	10	17
	2010	Mk5, 7 & 19	16	9	3	Yes (1)	17	14	4
	2011	Mk7 & 19	42	31	19	Yes (4)	71	2	7
	All years		119	75	39	8	155	30	46
Bird Island	2008	Mk7	29	3	2	No	6	24	-
	2009	Mk7	30	9	5	No	18	15	-
	2010	Mk7 & 19	30	10	5	No	21	9	-
	2011	Mk 19	30	6	4	No	11	46	-
	All years		119	28	16	No	56	94	-
**Total**			**238**	**103**	**55**	**8**	**211**	**124**	**46**

Between-year fidelity (YES or NO) indicates for which years multi-year animals were tracked with the number of individuals in each of those years in brackets.

^a^Refers to all trips that were undertaken by tracked animals (All), and the number of trips that could be used to compute utilisation distributions based on suitable minimum number of ARS locations (Fidelity level: within-year and between-year, see results).

^b^Between-year fidelity (YES or NO) indicates for which years multi-year animals were tracked with the number of individuals in each of those years in brackets.

### Study Site, Animal Handling and Instrumentation

**Fig 1:**
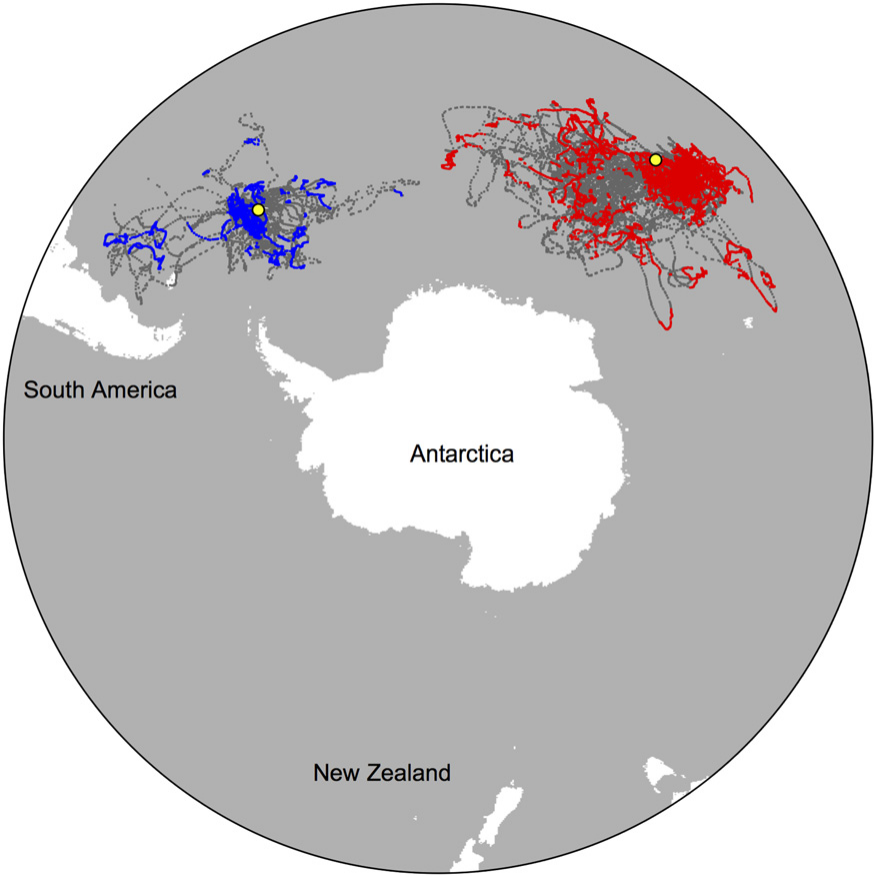
Mean estimated winter migrations for 59 adult female Antarctic fur seals from Bird Island and Marion Island, 2008–11. Locations in red and blue represent likely area-restricted search (ARS) behaviour for animals from Marion and Bird Island respectively, inferred through state space modelling. Colonies are shown in yellow.

The study took place on Marion Island (46°54’S, 37°44’E), Prince Edward Islands, southern Indian Ocean and Bird Island (54°00’S, 38°03’W), South Georgia, southern Atlantic Ocean between 2008 and 2011 (Fig. 1). Breeding adult female AFS were captured during the latter part of lactation (February to April) after they had dispersed from breeding harems. On restraint, individuals were instrumented with global location sensing (GLS) loggers to track at-sea position during their winter migrations (~8–9 months from April to December). Coloured plastic flipper tags (Dalton Supplies, Henley-on-Thames, UK) bearing a matching unique numeric sequence were inserted into the trailing edge of each fore-flipper [36]. The GLS loggers were first attached to a metal flipper tag using a two-part epoxy (Araldite K268, Ciba-Geigy Corp., Basel, Switzerland) and a plastic cable tie; this was then deployed on the fore flipper paired with one of the plastic flipper tags. Three models of GLS loggers manufactured by the British Antarctic Survey (BAS, Cambridge, UK) were deployed during the four-year study (Mk5 and Mk7–18 x 18 x 6.5 mm, 3.6 g and Mk19–16 x 14 x 6 mm, 2.5 g) (Table 1).

Seals were recaptured and their GLS loggers recovered at the beginning of the following austral summer (November to December) when pregnant females return to the colony to pup. Five animals were not recaptured until the end of the following winter and three individuals were tracked over three years (Table 2). As this study did not form part of a wider demographic enquiry, the age and reproductive success of tracked animals is unknown.

**Table 2:** Foraging trips per year and Utilisation Distribution overlap values (Bhattacharyya’s affinity) for eight female Antarctic fur seals from Marion Island that were tracked for multiple winters between 2008–2011.

Seal ID	Year				Total trips	Within year overlap^a^	Between year overlap
	2008	2009	2010	2011			
PP620	1	1	-	-	2	-	0.05
PP623	2	2	-	3	7	0.21	0.80
WB438	9	6	-	-	15	0.24	0.67
WB449	1	3	-	1	5	0.20	0.52
WB458	1	2	-	-	3	-	0.02
WB462	-	1	-	2	3	-	0.84
WB482	1	-	-	1	2	-	0.04
WW422	3	2	4	-	9	0.13	0.81
	2.6 ± 1.1	2.4 ± 0.6	-	1.8 ± 0.5	5.8 ± 1.6	0.2 ± 0.02	0.50 ± 0.08

^a^Animals with no within year overlap value either undertook only one trip per year, or successive trips were excluded from analyses as they contained fewer than 10 ARS locations (see Methods).

The loggers measured ambient light every minute and recorded the maximum value for every 10-minute period (5 minutes for Mk19 units). They also recorded sea temperature after 20 minutes continuous wet, repeated every 4–24.8 hours, and reset anytime the unit was dry for >3–6 seconds. Temperature was logged at a resolution of 0.125°C and with accuracy of ± 0.5°C, which was later improved by temperature calibration of each tag in a water bath [44]. The light loggers on each device were calibrated at each study site for approximately 5–7 days either immediately before or after deployment to obtain a solar elevation curve at a known locality, which was necessary for location estimation.

### Location Estimation

Location estimates were produced from the raw light and temperature data using the Bayesian approach of Sumner et al. [45] using the R package ‘tripEstimation’ [46] following the methodology detailed in Lea et al. [44]. In brief, the posterior mean for each twilight period (dawn and dusk) were summarized based on the accepted Markov Chain Monte Carlo (MCMC) samples, resulting in two location estimates per day. The accuracy of location estimates using this approach is shown to be 70 ± 35 km for an AFS carrying GLS and Argos tags simultaneously. [44]. Mean location estimates were used to facilitate the calculation of utilisation distributions (UD, see below), which would otherwise be computationally restricted if all MCMC estimates were considered. To ensure that UDs, and subsequent overlap, were not affected by this approach, a comparison was made with UDs calculated from a fixed number of accepted MCMC samples for a subset of animals (S1 Protocol). Furthermore, our state-space modelling approach also necessitated mean location estimates. State-space models built specifically for geolocation data were used to infer area restricted search (ARS) behaviour, indicative of probable large-scale foraging behaviour [47]. Model design and implementation closely followed the framework proposed by Jonsen et al. [48] and is described in detail in Lea et al. [44].

Individual trips were identified by examining the raw light data, with on-shore periods typified by obvious messy light curves caused by the animal periodically shading the light sensor during haul out. Each trip was analysed independently. Winter foraging trips were considered to be from the first post-weaning excursion (typified by a clear increase in trip duration when compared with shorter trips during lactation), to the return of the animal to the colony the following breeding season.

### Utilisation Distribution Estimation and Overlap

To assess habitat use, and the potential for overlap during winter foraging trips we calculated the 95% utilisation distribution (UD) using the fixed Kernel Density Estimation method derived from the least-squares cross validation bandwidth [49] in the R package ‘adehabitatHR’ [50]. Only locations associated with ARS behaviour, as indicated by the state-space models, were included in the analyses, meaning UDs represented an individual’s broad-scale foraging range rather than whether individuals simply followed the same migratory pathways. We computed the UD for individual animals to assess site fidelity at two scales: between years and within years (see Table 1):

1. *Between year site fidelity*—the UD was computed using all the ARS locations obtained for each year for those animals tracked over multiple winters.
2. *Within year site fidelity*—this was examined by calculating the UD based on ARS locations of individual foraging trips undertaken by each animal during a single year.

For all analyses, tracks with fewer than 10 locations were excluded as kernel estimation is robust above a minimum threshold of locations [51]. In some instances when UD models would not converge, a small amount of noise was introduced to location estimates using the “jitter” function (package ‘base’) to counter the high variance in estimates associated with spatially clustered locations [52] experienced for ARS locations. The amount of “jitter” introduced was never greater than the mean error surrounding the location estimates. UDs were estimated across a 1° raster grid encompassing the area 80°00’S—30°00’S; 140°00’W—00°00’E, to aid subsequent comparison with environmental variables.

Fieberg and Kochanny [53] undertook an extensive review of the indices of overlap between utilization distributions (UD), recommending Bhattacharyya’s affinity (BA) [54] for a general measure of similarity between UD estimates. BA considers the spatial domain of home ranges, ignoring their density of use, and estimates the percentage overlap between them when overlayed. We determine this an appropriate method as the primary interest of this study is the outright re-use of previous areas, rather than a finer scale assessment of home ranges. BA is given as a measure of affinity ranging from 0 (no overlap) to 1 (identical UDs) and was calculated using the “kerneloverlaphr” function in the ‘adehabitatHR’ package [50].

For a three or more way overlap, all trips/years were included and any grid cells that were used more than twice were considered to be overlapping, regardless of the degree of overlap.

### Environmental Variability

To investigate the role of environmental characteristics in influencing the degree of UD overlap for AFS, we extracted sea surface temperature (SST), sea surface height anomaly (SSHa) and chlorophyll a concentration (CHLa), from regions corresponding to UDs (Table 3). All available data were used and then restricted to the period of winter migrations (April—December). Data were first transformed to meet the assumptions of normality and we then calculated the mean and standard deviation (SD) of each parameter per pixel over the time period to create a temporal climatology [55], permitting an assessment of the long-term temporal patterns of variability [27].

**Table 3:** The source, timespan, spatial and temporal resolution and whether temporal climatologies were calculated for oceanographic data for comparison between overlapping and non-overlapping foraging regions.

Variable	Source	Frequency	Spatial resolution^a^	Timespan	Variance
SST—sea surface temperature	NOAA Optimum Interpolation daily Sea Surface Temperature^b^	5 days	0.25 degree	1988–2011	Yes
SSHa—sea surface height anomaly	AVISO^c^	7 days	1/3 degree (Mercator)	1999–2011	Yes
CHLa—chlorophyll a concentration	MODIS^d^	8 days	0.1 degree	2002–2011	Yes

^a^All data were reprojected into 1 degree pixels

^b^OI-daily: http://www.ncdc.noaa.gov/oa/climate/research/sst/oi-daily.php

^c^AVISO: http://www.aviso.oceanobs.com/en/data/products/sea-surface-height-products/global/index.html

^d^MODIS: http://oceancolor.gsfc.nasa.gov/

A comparison of environmental parameters within *non-overlapping areas* (cells used by an individual only once. i.e. year *j* for between-year fidelity, and trip *j* for within-year fidelity) and *overlapping areas* (grid cells used more than once i.e. year *j* + 1, and trip *j* + 1) was undertaken with logistic Generalised Linear Mixed Models (GLMMs) using the “lmer” function (package ‘lme4’). The response term (whether a grid cell was overlapping or non-overlapping) was fitted to a binomial error structure and logit-link function due to the binary nature of the response variable and the continuous nature of the predictor variables. Seal identity was included as a random effect when investigating between-year fidelity, whilst both seal identity and site were fitted as random effects when investigating within-year fidelity (all seals with multi-year tracks were from Marion Island, Table 1). Prior to model building, correlation between predictor variables was examined with a correlation matrix and Pearson product-moment correlation analyses were undertaken to quantify co-linearity. The distribution of predictor variables was also examined and data were log-transformed to meet the assumptions of normality where appropriate. Models were fitted using Laplacian approximation and were built from the null model to the saturated model considering all possible model combinations. Models were ranked using the AIC (Akaike Information Criterion), which includes the maximized log-likelihood of the model and penalises model complexity [56]. The best of the available models was determined using delta AIC and weights of evidence [57].

## Results

### Location Statistics and Track Summaries

We collected winter tracks for 103 adult female AFS from Marion Island (n = 75) [58] and Bird Island (n = 28) [59] between 2008–11. Multi-year tracks were available for eight individuals, all from Marion Island (N = 46 trips) and tracks of repeat trips within a year were available for 55 individuals (N = 124 trips), totalling 211 individual foraging trips and 33 716 location estimates, of which 15 295 (45%) were identified as likely ARS behaviour (Fig. 1). Four individuals completed multiple within and between-year trips, meaning the total number of animals used for analyses was 59. A detailed summary of sample sizes across the colonies and years is given in Table 1. Henceforth, all means are reported plus or minus standard error and all t-tests are two tailed. Among all individuals tracked, the mean maximum distance travelled from the colony was 1259 ± 56 km per trip (range 104–4528 km). The mean foraging trip duration was 123 ± 6 days (range 6–266 days) and the mean proportion of the trip spent in area-restricted search (ARS) behaviour was 41 ± 2% (range 1–96%).

### Foraging Site Fidelity

To determine if foraging areas were unique to individual seals we compared the overlap of UDs across all animals at each site. The mean inter-individual overlap of foraging home ranges was 0.14 ± 0.01 (range 0.01–0.28) at Marion Island and 0.22 ± 0.03 (range 0.01–0.38) at Bird Island. This indicates that individuals from these populations forage over a broad geographical range and that the overlap of foraging home ranges reported here is not merely a product of all animals moving to the same general area.

### Within-year fidelity

**Fig 2:**
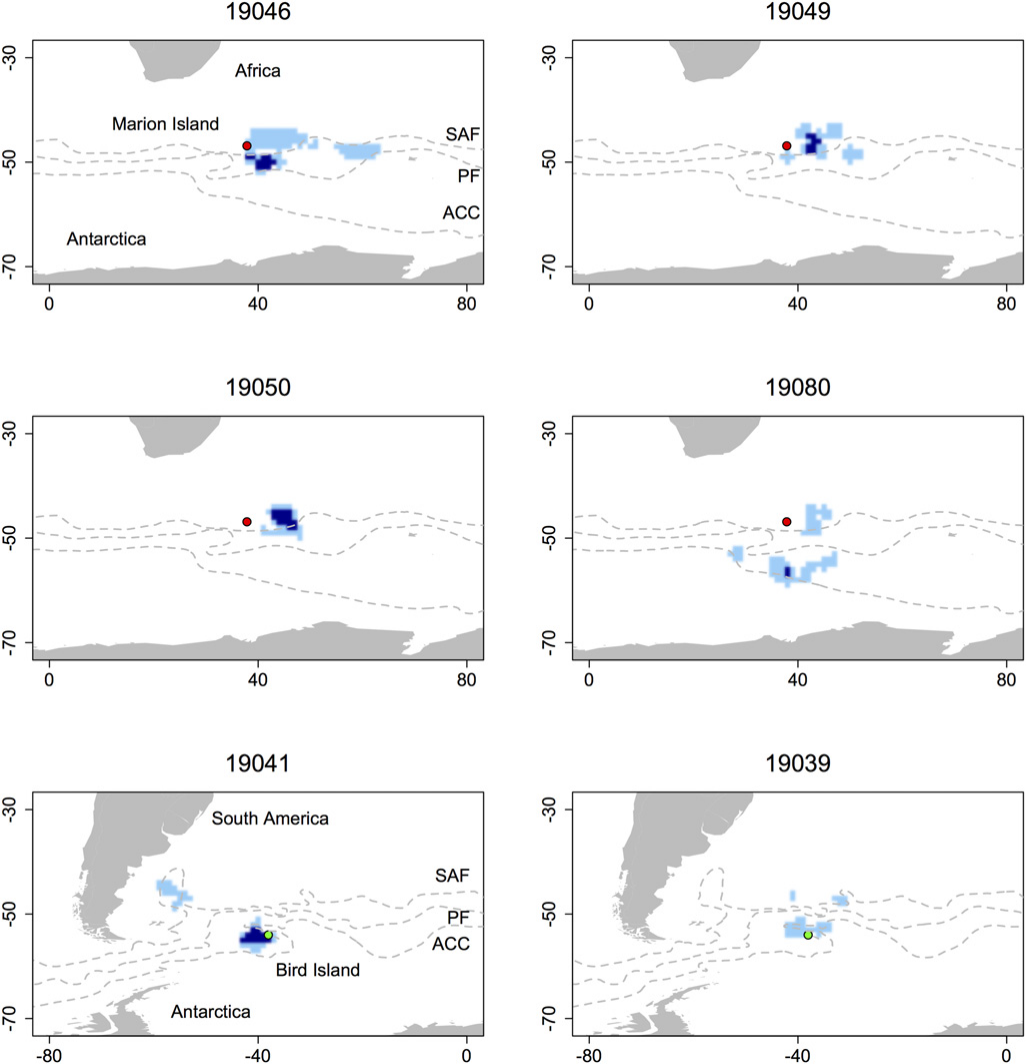
Within-year winter foraging habitat of six example adult female Antarctic fur seals. Light blue indicates area-restricted search (ARS) cells used during one trip only, while dark blue indicates overlapping cells used across multiple trips within a year. Lines indicate the mean location of the sub-Antarctic front (SAF), polar front (PF) and the Antarctic circumpolar current (ACC). Marion and Bird Island are shown in red and green respectively.

Thirteen trips were excluded from these analyses as they were either composed of fewer than 10 ARS locations, or would not converge during estimation of the UD. Therefore, 124 trips from 42 individuals were available, with individuals performing between two and nine repeat trips within a year. The mean size of UDs per trip was 23.4 ± 1.6 (range 4–136) 1^o^ grid cells. There was no difference in the mean size of UDs of trips from Marion (22.7 ± 1.8) and Bird Island (25.6 ± 3.2; t_46_ = -0.77, *P* = 0.445). Within individuals, the mean overlap of the foraging home range between successive trips was 0.15 ± 0.02 (range 0–0.81) at Marion Island and 0.21 ± 0.05 (range 0–0.74) at Bird Island. Across the two colonies, the mean within-year overlap of individual foraging home ranges was 0.16 ± 0.02 (range 0–0.80; Fig. 2).

### Between-year fidelity

**Fig 3:**
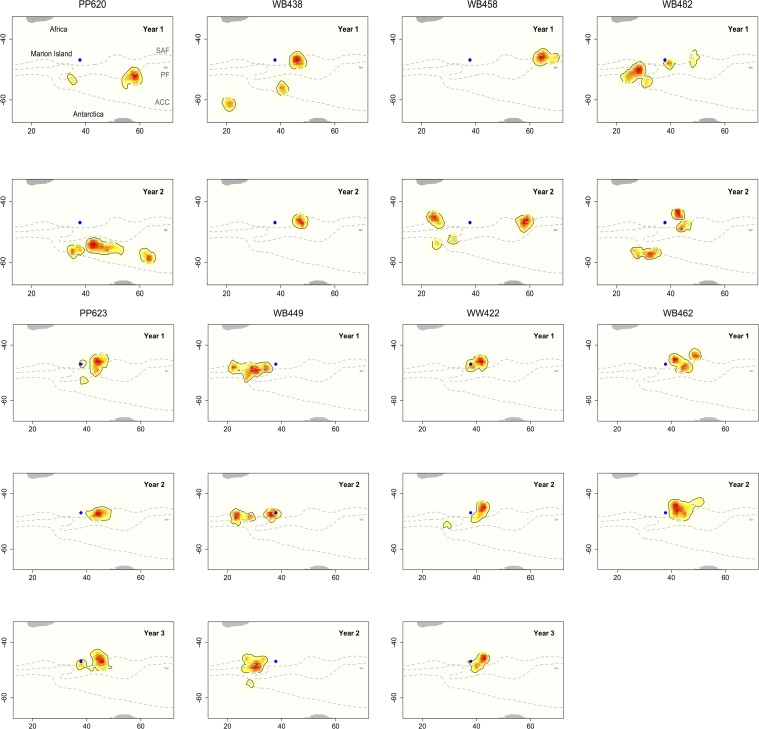
Utilisation distributions (UDs) for eight female Antarctic fur seals that were tracked over multiple winters. The black lines denote the 95% UD, which represents the annual foraging kernel home range of each animal. The individuals were tracked from Marion Island (blue circle) for either two or three years between 2008–2011. Grey lines show the mean position of the sub-Antarctic front (SAF), polar front (PF) and the Antarctic circumpolar current (ACC).

**Fig 4:**
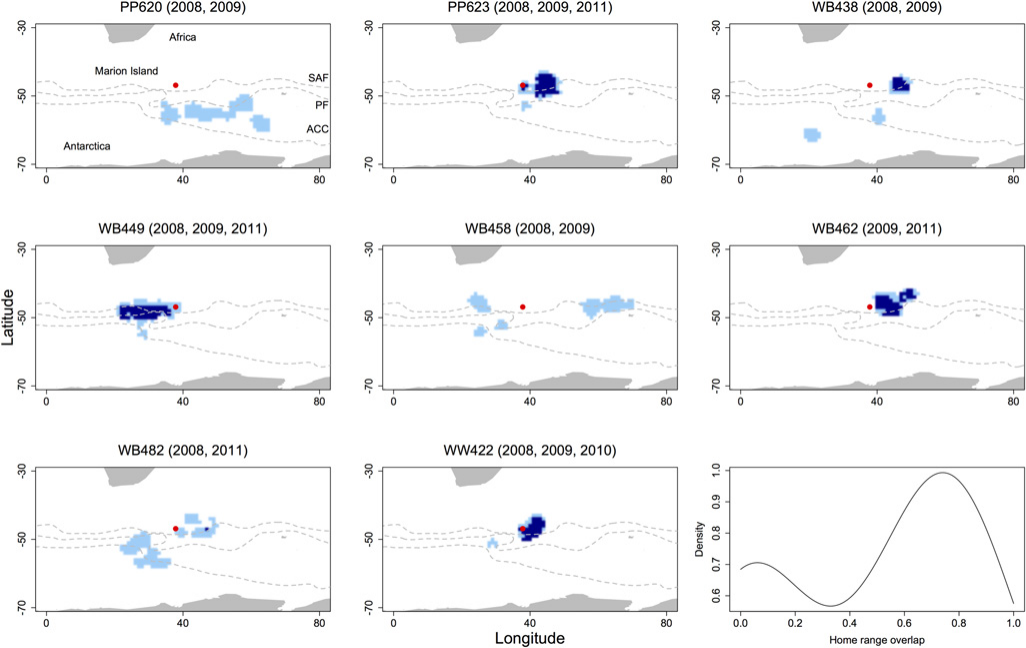
Multi-year foraging habitat use of eight female Antarctic fur seals from Marion Island during winter between 2008–2011. Light blue denotes cells used in one year, dark blue denotes overlapping cells used in multiple years. Lines indicate the mean location of the sub-Antarctic front (SAF), polar front (PF) and the Antarctic circumpolar current (ACC). The density distribution of home range overlap values (Bhattacharyya’s affinity) is shown in the bottom right panel.

A total of 4138 ARS locations were available for eight individual animals tracked over multiple years. Individuals were tracked for either two or three seasons and undertook between one and nine trips per season (Table 2). The mean size of UDs was 50.3 ± 3.9 (range 22–92) 1^o^ grid cells (Fig. 3). Within individuals, the mean home range overlap between years was 0.50 ± 0.08 (range 0.02–0.84; Fig. 4; Table 2). However, the degree of home range overlap within the sample population displayed an obvious bimodal distribution (Fig. 4), with three individuals having overlap values of 0.05 or less, while the five remaining individuals had overlaps of greater than 0.50 (Table 2). Overall, foraging home range overlap was significantly higher between years than within years, both when comparing across all animals (t_24_ = 3.96, *P* < 0.001) and animals from Marion Island only (t_23_ = 4.04, *P* < 0.001).

### Environmental Characteristics of High Use Regions

We compared environmental characteristics of individual foraging home ranges within and outside the overlap areas. Satellite-derived oceanographic parameters (sea surface temperature (SST), sea surface height anomaly (SSHa) and chlorophyll a concentration (CHLa) (Table 3) of the home ranges were examined. All data were re-projected into raster grids with a 1° resolution and a spatial extent of 80°S-30°S, 140°W-80°E. SSHa data was interpolated from the original 1/3 degree Mercator resolution. The long-term mean and standard deviation (SD) for the winter season for each grid cell over the region was calculated. After examination of these climatologies, we found there was poor temporal resolution of CHLa data during the winter period for many grid cells across the region, a common issue with satellite ocean colour products in the Southern Ocean caused by reduced temporal and spatial coverage corresponding to increased cloud cover at this time of year [60]. CHLa data was therefore excluded from further analyses to ensure all climatologies were calculated from a consistent minimum number of data points across the spatial domain.

### Regions of within-year overlap

**Fig 5:**
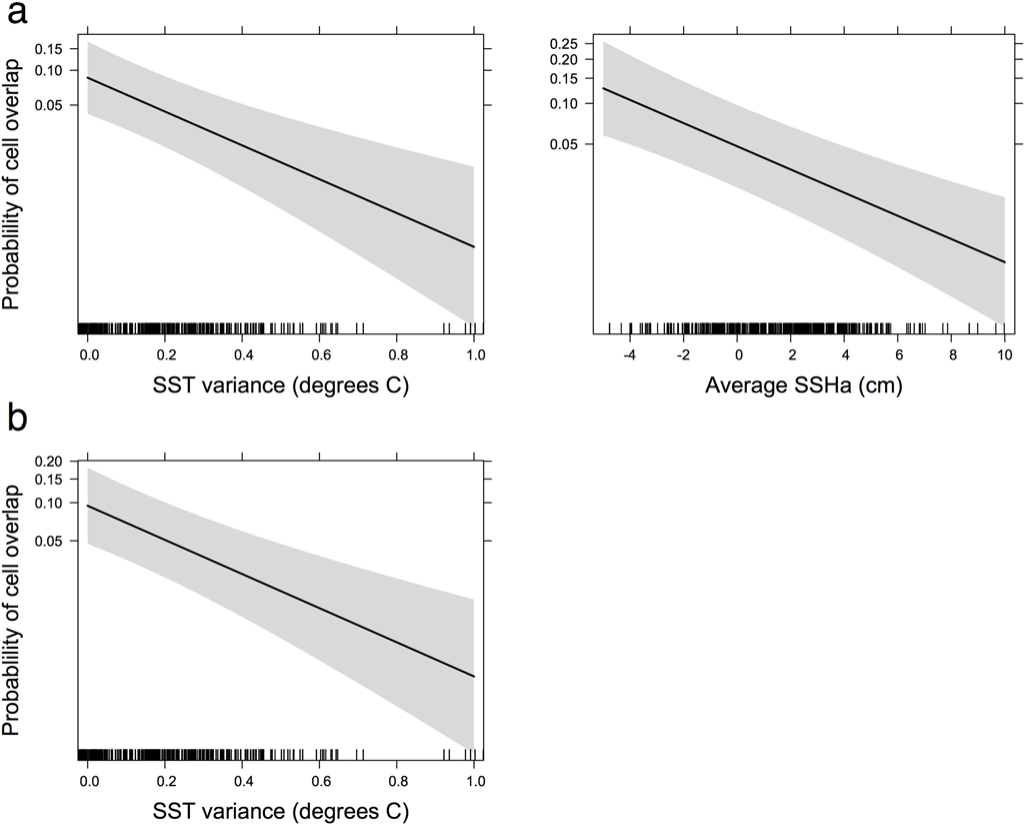
Probability of foraging site fidelity in relation to oceanographic parameters: (a) within a year and (b) between years. Curves were fitted using the best logistic GLMM respectively, as shown in Table 5. The grey bar represents the 95% confidence interval around the estimated effect.

We compared the environmental climatologies of regions of home range overlap between successive foraging trips within a year, with non-overlapping regions visited during one trip only. A Pearson product-moment correlation analysis indicated co-linearity between SST_mean and SST_SD (*r*_1335_ = 0.58, *P* < 0.001). SST_mean was therefore removed from the analyses as we were interested in the effects of long-term environmental variability on site fidelity and the SST_SD (a measure of variance) is a more relevant variable. The best model regarding whether a grid cell was overlapping or non-overlapping (termed ‘*celluse*’ in the model) included SST_SD and SSHa_mean (AIC weight = 0.593; model 1 Table 4a). A subsequent test for an interaction effect between the fixed predicator terms by including this in the model resulted in a poorer model performance (**Δ**AIC = 1.2; model 2 Table 4a). Based on the accepted model (model 1) the probability that grid cells would overlap across successive trips within a particular year increased for cells with lower SST_SD and negative SSHa_mean (Table 5a, Fig. 5a).

**Table 4:** Summary of generalised linear mixed-effect model (GLMM) comparisons: (a) GLMMs of cell use for within-year fidelity include seal identity and site as random effects; and (b) GLMMS of cell use for between-year fidelity include seal identity as a random effect (“celluse” = overlapping or non-overlapping, SST_SD = sea surface temperature standard deviation, SSHa_mean = average sea surface height anomaly, SSHa_SD = sea surface height anomaly standard deviation).

Candidate models	*k*	LL	AIC	ΔAIC	wAIC
(a) *GLMMs of oceanographic parameters—within year*					
**1. celluse ~ SST_SD + SSHa_mean**	**5**	**-413.8**	**837.7**	**0.0**	**0.593**
2. celluse ~ SST_SD + SSHa_mean + SST_SD*SSHa_mean	6	-413.4	838.8	1.2	0.331
3. celluse ~ SSHa_mean + SSHa_SD	5	-415.9	841.9	4.2	0.071
(b) *GLMMs of oceanographic parameters—between year*					
**1. celluse ~ SST_SD**	**3**	**-185.7**	**377.4**	**0.0**	**0.366**
2. celluse ~ SST_SD + SSHa_SD	4	-185.0	377.9	0.5	0.288
3. celluse ~ SST_SD + SSHa_mean	4	-185.3	378.6	1.2	0.202
4. celluse ~ SSHa_mean + SSHa_SD + SST_SD	5	-184.7	379.3	1.9	0.142

Only models with a delta AIC <10 are presented and the accepted model is presented in bold.

*k*, number of paramaters; LL, log-likelihood; AIC, Akaike’s Information Criterion; **Δ**AIC, difference in AIC from that of the best fitting model; *w*AIC, AIC weight.

**Table 5:** Results for the best available generalized linear mixed-model (GLMM) examining the effects of oceanographic parameters on (a) within-year foraging site fidelity and (b) between-year foraging site fidelity of female Antarctic fur seals.

Parameter^a^	Variance	Estimate	SE	95% CI
(a) *Within-year*				
*Fixed*				
Intercept		-2.363	0.364	
SST_SD		-2.566	0.777	-4.09, -1.64
SSHa_mean		-0.211	0.045	-0.30, -0.12
*Random*				
Seal ID	3.078			
Site	0.000			
N_cells_ = 1337			N_seals_ = 42	
(b) *Between-year*				
*Fixed*				
Intercept		-3.187	1.188	
SST_SD		6.899	1.433	4.08, 9.71
*Random*				
Seal ID	9.753			
N_cells_ = 580			N_seals_ = 8	

The best of the available models was determined using delta AIC and weights of evidence.

^a^SST_SD, sea surface temperature standard deviation; SSHa_mean, average sea surface height anomaly; N_cells_, number of grid cells; N_seals_, number of individual seals; SE, standard error; 95% CI, 95% confidence interval.

### Regions of between-year overlap

The environmental variables in regions of annual foraging home range overlap were compared with non-overlapping regions used in a single year only. The best model explaining cell overlap included SST_SD (AIC weight = 0.366; model 1 Table 4b). We found the probability that grid cells would overlap between years increased significantly for cells associated with higher variance in SST (Table 5b; Fig. 5b).

## Discussion

Most studies of foraging behaviour seek to identify aspects of foraging strategies, such as habitat preference or prey searching techniques, with little consideration of whether particular strategies are consistent over time. It is often unknown if behaviours observed in one time period (i.e. one season or one foraging trip) are an accurate representation of an individual’s longer-term foraging behaviour. This is true of many animal tracking studies, where we often do not know if locations from one year are indicative of a stable, long-term foraging strategy [31]. As foraging behaviour can vary in response to a multitude of factors including prey availability and distribution, environmental conditions, competition and the energetic requirements associated with age and breeding status [61–65], it is important to identify the time-scale over which these behaviours persist. Our results showed that female AFS utilise a wide range of foraging habitats during the non-breeding winter season, with high levels of individual variation in foraging area as indicated by relatively low inter-individual foraging range overlap. Most individuals, however, displayed some degree of site fidelity to foraging areas, particularly over the mid to long term (i.e. between years).

When estimating the overlap of individual foraging areas the choice of scale will inevitably affect the results. Too fine a scale may yield little or no overlap, while high levels of overlap may result at coarser scales. Assessing the overlap of UDs, which provide a practical summary of space use for a given individual [53], overcomes these issues. We calculated UDs across a 1° grid, chosen to aid comparison with environmental data and match the error uncertainty surrounding location estimates via geolocation (70 ± 35 km) [44]. As kernel density estimates are largely unaffected by grid size [52], the resolution of this grid does not have a significant impact on the estimates of UDs and their resulting overlap. The estimation of kernel based UDs are less accurate for small samples [66] and it is therefore possible that the lower overlap values reported within years are partly an artefact of fewer foraging locations from shorter trips. However, by excluding very short foraging trips (<10 ARS locations) from our analyses we are confident that our results are spatially robust.

Female AFS displayed strong individual foraging site fidelity between years. On average, seals utilised 50% (± 8% SE) of their overall foraging range across multiple years. Multi-year foraging site fidelity has been reported for few marine taxa including turtles [32, 33] and rays [67] and has also been noted in cetaceans mostly through re-sight studies [19, 68]. Multi-year fidelity to foraging sites has been described in only a handful of pinniped species. Both Chilvers [31] and Augé et al. [34] showed that individual female New Zealand sea lions (*Phocarctos hookeri*) displayed strong site fidelity across two years, with, on average a 64% inter-annual overlap of home ranges during short autumn trips [34]. Bradshaw et al. [27] also reported strong overlap in the habitat use of female southern elephant seals (*Mirounga leonina*) during post lactation (66%) and post moult (53%) trips from Macquarie Island. Using a different approach, Lowther et al. [69] report broad scale multi-year site fidelity in Australian sea lions (*Neophoca cinerea*) using stable isotope analysis, with individuals consistently exploiting either inshore or offshore sites. Unlike New Zealand and Australian sea lions, which are typically benthic foragers that undertake short, repeat trips of several hundred kilometres [34], AFS (and southern elephant seals) can undertake wide-ranging foraging migrations of many thousands of kilometres [35, 36]. During this time, animals are exposed to a range of environmental conditions and are likely to be making judicious choices regarding foraging habitat selection. The eight animals tracked over multiple years in this study displayed a range of overlap values, with less than 5% overlap between years for three individuals, while five individuals recorded overlap values of greater than 50%, suggesting a bimodal strategy of foraging site fidelity among individuals. Precisely what drives these different strategies is difficult to say. However, we note that all animals displaying a low degree of site fidelity undertook a single foraging trip in each year, while animals that were highly faithful to foraging sites undertook at least two repeat trips throughout the years they were tracked.

We show that areas of multi-year overlap were not stable, but rather highly variable. Individual AFS that were tracked over multiple years displayed greater fidelity to areas characterised by a high variance in SST over multiple decades, with the probability that a cell would be used in multiple years higher for cells that exhibited greater long-term variability in SST, a potential proxy for long-term productivity. This is similar to southern elephant seals [27], which also returned to regions with higher long-term variance in SST, perhaps because these areas yield a higher prey abundance. Indeed the greater variability of SST within frontal regions of the Southern Ocean is often correlated with elevated productivity when compared with surrounding areas [70]. We may consider such areas to be of higher habitat quality and, therefore, the target of foraging animals. While there is some degree of spatial predictability in the structure of major frontal regions in the Southern Ocean [71, 72], the position of fronts varies between years [73], making habitat quality less spatially and temporally predictable. Based on our sample size of eight seals, some individuals foraging in such variable environments appear to settle on a territory over the long-term. As an hypothesis for further study we suggest that this strategy will function to maximise net energy gain, and therefore fitness, over the long-term i.e. the individual’s lifetime [26] and will seemingly persist regardless of annual variations in energy intake. The wide geographical spread of areas of foraging overlap observed among individuals indicates that these results are not simply a product of all individuals targeting the same broad areas and that longer-term fidelity to foraging regions is a more prevalent behavioural mechanism than offsetting changing prey resources by shifting to alternate foraging habitats.

Within an annual cycle, fidelity to foraging sites was much weaker with, on average, 16% (± 2% SE) of an individual’s foraging area utilised across multiple trips. A similar level of intra-annual site fidelity (13% overlap) was reported in the foraging routes of lactating New Zealand fur seals (*A*. *forsteri*) [74], while others have suggested greater intra-annual site fidelity in pinnipeds [16, 75], including AFS [42]. These studies, however, focussed on lactating females which have short, constrained foraging trips compared with the winter migrations reported here, and were based on directional fidelity rather than overlap of the individual’s total foraging range, which are likely to produce greater estimates of fidelity. Using this method, Bonadonna et al. [42] concluded that individual AFS learn the broad direction of travel to a profitable area, but during a trip they forage opportunistically whenever good patches are encountered. While seals may exploit areas of previous foraging success during subsequent trips, Staniland et al. [41] suggest this occurs when patches are stable both spatially and temporally. The weaker foraging site fidelity within years reported in this study supports these findings and may be driven, in part, by seasonal shifts in ocean conditions. The results of the best-fit GLMM indicated that non-overlapping areas that were visited during one trip only, which was the dominant within-year strategy, were typified by high variability in SST. The habitat quality of these areas is likely to be less stable when compared with areas of low variance, yet may be associated with increased foraging habitat quality at particular times of the year.

We suggest that individual AFS display directional fidelity towards profitable regions within a year, as proposed by Bonadonna et al. [42], on a broad to meso-scale, where they search for prey that are ephemeral at short-term temporal scales, which drives increased habitat switching. The ‘win stay/lose switch’ rule [76] seems applicable, where individual AFS will show greater fidelity if more successful during previous trips, but once success decays, presumably driven by a reduction in habitat quality with changing environmental conditions, animals wont return and instead search for more profitable areas. This strategy agrees with classic foraging ecology models such as the Marginal Value theorem [1], explaining how an individual will forage in a predictably patchy environment. It is difficult to infer what oceanic features the individuals adopting this strategy are targeting (if any), however, areas visited on one trip only were characterised by positive SSH anomalies. Such conditions may be indicative of short-lived meso-scale, warm-core eddy features [77] which are associated with enhanced phytoplankton productivity [78] and known to be the target of foraging marine predators [79], specifically from Marion Island [80].

There may be several benefits to those individual AFS that favour foraging site fidelity as a strategy, particularly over the long-term. Ultimately, it is a behavioural adaptation, involving consideration of both prey richness and predictability, which should minimise energy expenditure while maximising net energy gain. The underlying driver of this benefit may be rooted in spatial familiarity, where prior knowledge of an area leads to heightened individual fitness because of increased foraging effectiveness [81], reduced predation risk and/or reduced travel costs [23]. Furthermore, foraging site fidelity will probably strengthen with age, as there will be fewer reproductive events available to compensate for the potential costs associated with switching habitat [5]. Authier et al. [82] showed that a stable foraging strategy developed earlier in life corresponded with increased longevity in male southern elephant seals. The development of foraging site fidelity in AFS may be the result of initial success as a juvenile, where the productive foraging routes learned during early foraging trips [83] persist into adulthood. Ideally, we would quantify the success of foraging site fidelity as a behavioural adaptation through demographic measures such as longevity or breeding success. Behavioural strategies that operate at the individual level, such as fidelity to foraging sites, have evolutionary and ecological implications and are potential drivers of niche specialization and intra-specific competition [84]. Furthermore, the strong multi-year site fidelity demonstrated in this study raises questions about the ability of long-lived animals such as pinnipeds, to respond to future environmental change.

## Supporting Information

S1 Protocol Effects of the Bayesian location estimation procedure on Utilisation Distributions and subsequent overlap. (DOCX)
