# Correction: Return Customers: Foraging Site Fidelity and the Effect of Environmental Variability in Wide-Ranging Antarctic Fur Seals

**DOI:** 10.1371/journal.pone.0179322

**Published:** 2017-06-06

**Authors:** Benjamin Arthur, Mark Hindell, Marthan Bester, Phil Trathan, Ian Jonsen, Iain Staniland, W. Chris Oosthuizen, Mia Wege, Mary-Anne Lea

In Fig 5, panel B is a duplicate of the first image of panel A. Please see the correct Fig 5 and its caption below.

**Fig 5:**
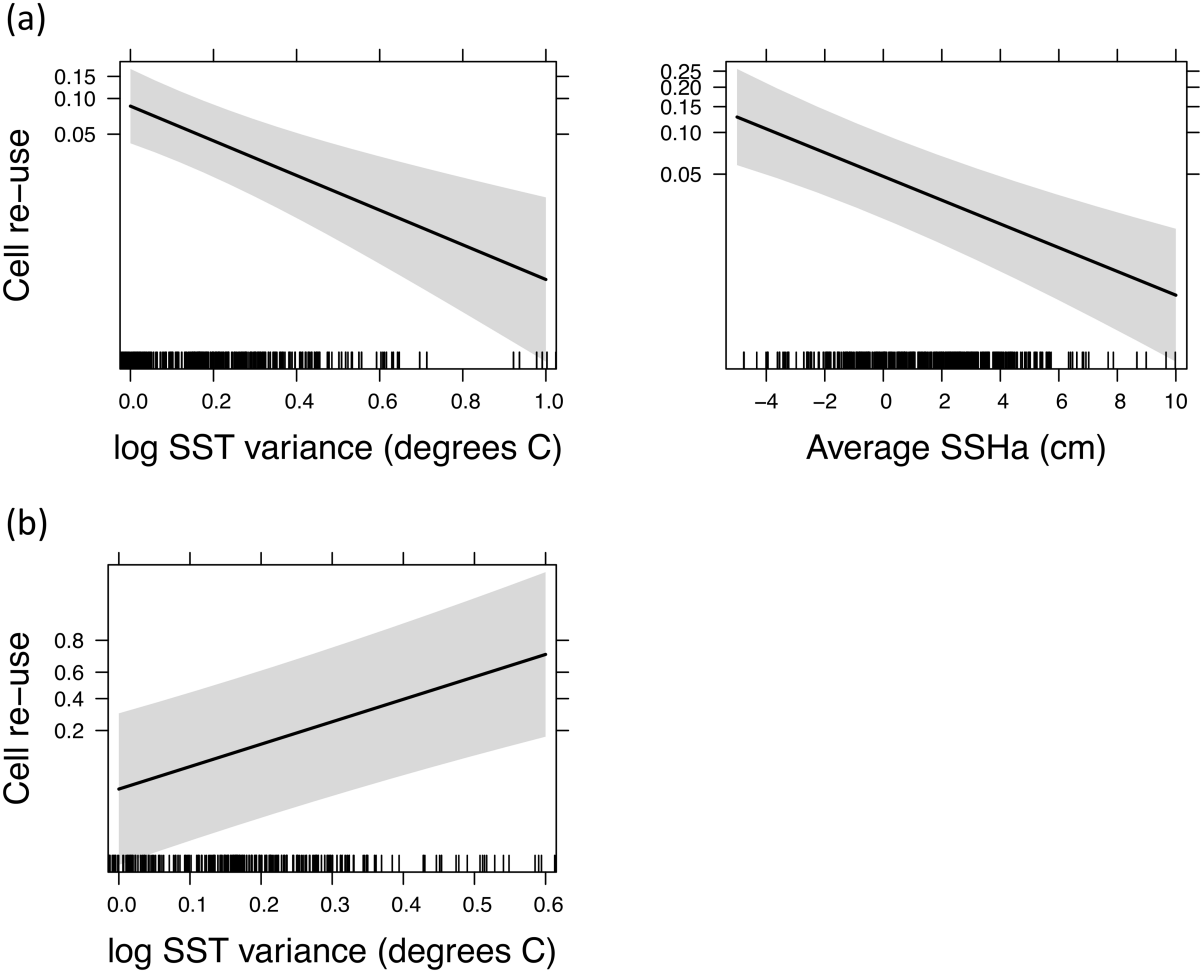
Probability of foraging site fidelity in relation to oceanographic parameters: (a) within a year and (b) between years. Curves were fitted using the best logistic GLMM respectively, as shown in Table 5. The grey bar represents the 95% confidence interval around the estimated effect.
