# Supplementary material for: Return Customers: Foraging Site Fidelity and the Effect of Environmental Variability in Wide-Ranging Antarctic Fur Seals
Source: PLoS One. 2015 Mar 25;10(3):e0120888. doi: 10.1371/journal.pone.0120888 (PMC4373865; doi:10.1371/journal.pone.0120888)
Supplement: S1 Protocol — (DOCX) [file pone.0120888.s001.docx]

**Protocol S1. Effects of the Bayesian location estimation procedure on Utilisation Distributions and subsequent overlap.**

To ensure that the Bayesian location estimation approach did not impact the degree of overlap of UDs (i.e. foraging home ranges) we undertook a comparison between our approach (summarizing the MCMC samples to obtain a posterior mean) and using a fixed number of accepted MCMC samples for each twilight. We generated UDs from the ARS locations and compared their overlap between these two approaches for a subset of eight animals, all of which undertook multiple foraging trips.

Using our original approach, the mean within individual overlap for these animals was 0.14 ± 0.03 SE (range 0-0.23). In our comparison, for each ARS location estimate we re-imposed 200 points regularly sampled from the MCMC approximations. These inflated location estimates were then used to generate UDs for overlap comparison, which is a computationally intensive process and not practical for the full data set. In this instance, the mean within individual overlap was 0.13 ± 0.03 SE (range 0-0.22). There was no significant difference in the overlap of foraging home ranges between these two approaches (t _7_ = 1.27, *P* = 0.252).
